# hsa_circ_0003176 Suppresses the Progression of Non-Small-Cell Lung Cancer via Regulating miR-182-5p/RBM5 Axis

**DOI:** 10.1155/2022/8402116

**Published:** 2022-09-23

**Authors:** Fangfang Yang, YanLi Pei, Wei Xu, Lei Rong

**Affiliations:** Department of Respiratory and Critical Care Medicine, The University of Hong Kong-Shenzhen Hospital, Shenzhen 518053, China

## Abstract

**Background:**

Non-small-cell lung cancer (NSCLC) is one of the major diseases that threaten human health, and there is still no fundamental treatment method. Emerging evidences suggested that circRNAs might be an effective target to treatment NSCLC. However, the roles and detailed mechanisms of hsa_circ_0003176 in NSCLC still not clear.

**Methods:**

hsa_circ_0003176 was identified from GSE101684 and GSE112214 datasets of Gene Expression Omnibus (GEO) database. The expression of hsa_circ_0003176 was detected by RT-qPCR in NSCLC tissues, paired adjacent nontumor tissues, and cell lines. RNA fluorescence in situ hybridization and nuclear and cytoplasmic RNA fractionation analysis was used to detect the subcellular localization of hsa_circ_0003176 in H1299 and A549 cells. Dual-luciferase reporter and RNA pull-down assay were used to confirm the regulatory of miR-182-5p to hsa_circ_0003176 and RBM5. The roles of hsa_circ_0003176 in NSCLC progression was evaluated both in vitro by CCK-8 assay, colony formation assay, wound-healing assay, and matrigel transwell assay and in vivo by the subcutaneous xenograft nude mouse experiment and lung metastasis nude mouse experiment. In addition, RNA pull down and luciferase reporter assays were carried out to investigate the interaction between hsa_circ_0003176 or RBM5 and miR-182-5p.

**Results:**

Our results indicated that hsa_circ_0003176 showed typical characteristic of circRNAs, which was downregulated in both NSCLC tissues and cell lines. Functionally, overexpression of hsa_circ_0003176 suppressed the proliferation, migration, and invasion of NSCLC cells in vitro and inhibited NSCLC growth and metastasis in vivo. Furthermore, we found that hsa_circ_0003176 acts as sponge of miR-182-5p to regulate RBM5 expression. Further, in vitro rescue experiments demonstrated that hsa_circ_0003176 suppressed the proliferation, migration, and invasion of NSCLC cells by regulating miR-182-5p/RBM5 axis.

**Conclusion:**

We demonstrated that hsa_circ_0003176 suppressed the NSCLC progression via regulating miR-182-5p/RBM5 axis. These data indicated that hsa_circ_0003176 might be a novel molecular target for NSCLC treatment.

## 1. Introduction

Lung cancer is the malignant tumor, which presented the highest morbidity and mortality all over the world [1]. Based on the pathological types, it is divided into small-cell lung cancer (SCLC) and non-small-cell lung cancer (NSCLC). Among them, NSCLC is characterized by slow growth and division, late metastasis and spread, and low malignancy, accounting of about 85% of the total number of lung cancers [2]. More than half of the patients are in the middle and late stages when they are diagnosed, losing the best time for treatment [3]. In recent years, important breakthroughs have been made in the treatment of NSCLC, which has developed from traditional surgery, chemotherapy, and radiotherapy to the era of precise molecular targeted therapy and immunotherapy [4]. However, the 2-year relative survival rate of NSCLC patients reached only 42% [5]. Therefore, in-depth clarifying the molecular mechanisms of the occurrence and development of NSCLC is crucial for the development of new antitumor drugs and the improvement of prognosis.

Circular RNAs (circRNAs) are a new class of noncoding RNAs and widely exist in the cytoplasm of eukaryotic cells [6]. circRNAs have a unique closed-loop structure and play a crucial role in regulating gene transcription, which can either directly affect protein expression, or act as ceRNAs to regulate target gene expression by sponging miRNAs [7]. Accumulating evidence has suggested that circRNAs not only play an important biological role in the development of organisms but also play a critical role in the diagnosis and treatment of different kinds of diseases [8, 9]. With the deepening of NSCLC research, increasing circRNAs proved to contribute to different processes of NSCLC. For example, Sun et al. identified 8 key circRNAs by comprehensive bioinformatics analysis with three gene expression microarrays [10]. Fu et al. indicated that hsa_circRNA_012515 was significantly increased in NSCLC patients and cells, indicating that it might be a potential biomarker in the prognosis of NSCLC [11]. Liang et al. showed that circRNA_103615 promoted NSCLC cisplatin resistance and tumor progression by regulating ABCB1 [12]. circNDUFB2 activated antitumor immunity that inhibited NSCLC progression by regulating IGF2BPs expression [13]. hsa_circ_0000429 was highly expressed in the NSCLC tissues and cell lines, and overexpression promotes NSCLC progression by elevating SMADD expression by sponging miR-1197 [14]. These findings suggested that exploring the roles and molecular mechanism of circRNAs in NSCLC is helpful for the identification of new molecular targets and the implementation of new treatment options for NSCLC.

Although current studies have demonstrated that circRNAs play an important role in the pathogenesis of NSCLC, the biological functions and regulatory mechanisms of most circRNAs in NSCLC remain still unknown and need to be further explored. Hereby, we identified that hsa_circ_0003176 (a circRNA derived from TBX5) was significantly downregulated in NSCLC tissues compared with match adjacent nontumorous tissues through analyzing the public lung cancer dataset in the GEO database and further to explored the biological function and underlying molecular mechanism of hsa_circ_0003176 in the development of NSCLC. Our study not only disclosed a novel mechanism of NSCLC development but also provided a new molecular target for NSCLC treatment.

## 2. Materials and Methods

### 2.1. Clinical Specimen

A total of 10 NSCLC tissues and 10 corresponding adjacent nontumor tissues were collected from the University of Hong Kong-Shenzhen Hospital (Shenzhen, China). These tissue specimens were reviewed and approved by the Committees for Ethical Review of Research at the University of Hong Kong-Shenzhen Hospital (approval number: [2020]171).

### 2.2. Data Sources and Bioinformation Analysis

The circRNA expression profile related to NSCLC was investigated in GSE101684 and GSE112214 data sets from GEO database (https://www.ncbi.nlm.nih.gov/gds/). Receiver operating characteristic (ROC) curve assay was used to detect the area under curve (AUC) of sensitivity and specificity. circBase (http://www.circbase.org/) and circRNADb databases (http://reprod.njmu.edu.cn/cgi-bin/circrnadb/circRNADb.php) were used to investigate the localization and the transcript of hsa_circ_0003176. The potential binding miRNAs of hsa_circ_0003176 were predicted by CircInteractome (https://circinteractome.irp.nia.nih.gov/). The target genes of miR-182-5p were predicted by PITA (http://genie.weizmann.ac.il/pubs/mir07/mir07_data.html), miRmap (https://mirmap.ezlab.org/), miRanda (http://bioinfo.au.tsinghua.edu.cn/micrornadb/index.php), and TargetScan databases (http://www.targetscan.org/). The relationship between circRNA and RBM5 was also confirmed by using the GEPIA (Gene Expression Profiling Interactive Analysis) online software (http://gepia.cancer-pku.cn/).

### 2.3. Cell Lines, Culture, and Treatment

Human normal bronchial epithelioid cells, 16HBE cells, and H1299, H446, 95-D, and A549 cell lines were purchased at the American Tissue Culture Collection (ATCC). Among H446 cell line is small-cell lung cancer cells, whereas H1299, 95-D, and A549 cell lines are non-small-cell lung cancer cells. These cell lines were cultured in DMEM (Gibico, USA) supplemented with 10% fetal bovine serum (FBS, Gibico, USA) and double antibiotic (100 IU/mL penicillin and 100 *μ*g/mL streptomycin) at 37°C in a humidified atmosphere with 5% CO_2_. The overexpression plasmid plenti-ciR-GFP-T2A-puro for hsa_circ_0003176 and the short hairpin RNA (shRNA) of RBM5 were designed and constructed to pLKO.1-U6 vector. The sequence of full length of hsa_circ_0003176, the shRNA of RBM5, miR-182-5p mimics, miR-182-5p inhibitor, and their corresponding negative control (NC) were synthesized at Sangon Biotech (Shanghai, China). All the plasmid or miRNAs mimics/inhibitor were transfected to the cells using Lipofectamine 8000 reagent (Beyotime, China) according to the protocols provided by the manufacturer. The sequence of all shRNAs, miR-182-5p mimics, miR-182-5p inhibitor, and their negative control is as follows: sh-RBM5#1: 5′-CCGGTCCCAGACCTAAGTTTGAAGATCTCGAGATCTTCAAACTTAGGTCTGGGTTTTTGAATT-3′, sh-RBM5#2: 5′-CCGGTACCATCACAGAGAGCGATATTCTCGAGAATATCGCTCTCTGTGATGGTTTTTTGAATT-3′, sh-RBM5#3: 5′-CCGGTCGCGTCTTTAGCTGTCAATAACTCGAGTTATTGACAGCTAAAGACGCGTTTTTTGAATT-3′, sh-NC: 5′-CCGGTCCTAAGGTTAAGTCGCCCTCGCTCGAGCGAGGGCGACTTAACCTTAGGTTTTTGAATT-3′, NC mimics: sense: 5′-UUGUACUACACAAAAGUACUG-3′ and antisense: 5′-GUACUUUUGUGUAGUACAAUU-3′, hsa-miR-182-5p mimics: sense: 5′-UUUGGCAAUGGUAGAACUCACACU-3′ and antisense: 5′-UGUGAGUUCUACCAUUGCCAAAUU-3′, NC inhibitor: 5′-CAGUACUUUUGUGUAGUACAA-3′, and hsa-miR-182-5p inhibitor: 5′-AGUGUGAGUUCUACCAUUGCCAAA-3′.

### 2.4. Quantitative Real-Time Polymerase Chain Reaction (RT-qPCR)

The total RNAs from cells and tissues were extracted using TRIzol reagent (Invitrogen, USA). For hsa_circ_0003176 and RBM5 expression detection, complementary DNA (cDNA) transcription from RNA was performed using PrimeScript™ RT reagent Kit with gDNA Eraser (Takara, RR047A). For miR-182-5p expression, the RNA was reversed transcribed into cDNA by miRNA 1st Strand cDNA Synthesis Kit (MR101-01, Vazyme). The Real-Time qPCR was conducted on LightCycler®96(Roche) to detect the mRNA abundance of hsa_circ_0003176 and RBM5 with SYBR Green qPCR Mix (D7260, Beyotime) and the miR-182-5p with miRNA Universal SYBR qPCR Master Mix (MQ101-01, Vazyme) according to the manufacturer's instructions. For the hsa_circ_0003176 identification by RT-qPCR, the total RNA was pretreated with 3 U/*μ*g RNase R enzyme for 20 min at 37°C. Glyceraldehyde-3-phosphate dehydrogenase (GAPDH) acts as internal control for hsa_circ_0003176 and RBM5, and U6 acts as internal control for miR-182-5p. Relative expression of genes was measured by the 2^-△△Ct^ method. The primer sequences used in RT-qPCR in the present study are as follows: hsa_circ_0003176: forward primer: 5′-GGAGCTGCACAGAATGTCAA-3′ and reverse primer: 5′-CTTTTGCGTCAGGCTCCAG-3′; divergent primer: forward: 5′-GGAGCTGCACAGAATGTCAA-3′ and reverse: 5′-CTTTTGCGTCAGGCTCCAG-3′; convergent primer: forward primer: 5′-ACGTCTTTCCTGAGACTGCG-3′ and reverse primer: 5′-TCATCACTGCCCCGAAATCC-3′; miR-182-5p: RT primer: 5′-GTCGTATCCAGTGCGTGTCGTGGAGTCGGCAATTGCACTGGATACGACAGTGTGA-3′, forward primer: 5′-CGGCTTTGGCAATGGTAGAAC-3′, and reverse primer: 5′-GTCGTATCCAGTGCGTGTC-3′; RBM5: forward primer: 5′-GACCGATCCGAAGATGGCTAC-3′ and reverse primer: 5′-TTGCTCTCCCTCTCGTCACTG-3′; GAPDH: forward primer: 5′-ACAACTTTGGTATCGTGGAAGG-3′ and reverse primer: 5′-GCCATCACGCCACAGTTTC-3′; and U6: forward primer: 5′-CTCGCTTCGGCAGCACATATACT-3′ and reverse primer: 5′-ACGCTTCACGAATTTGCGTGTC-3′.

### 2.5. RNA Fluorescence In Situ Hybridization (RNA FISH)

The cell slides were fixed in 4% paraformaldehyde for 20 min and washed 3 times in phosphate buffered solution (PBS, pH 7.4) on a destaining shaker. The cells were digested by added proteinase K (20 *μ*g/ml) for 5 min and then washed with PBS for 3 times. And then, the slides were incubated for 1 h at 37°C incubator with prehybridization solution. Subsequently, the hybridization solution containing the hsa_circ_0003176 probe with concentration at 1 *μ*M was added and hybridize at 42°C overnight. Then, the hybridization solution was washed with 2 × SSC, 1 × SSC, and 0.5 × SSC for 10 min, 5 min, and 10 min at 37°C, respectively. Specific probes of hsa_circ_0003176 (sequence: 5′-GCAAGGTTCTGCTCTTTGCAT-3′) were synthesized at Geneseed Biotech (Geneseed Biotech, Guangzhou, China). The immunofluorescence signal was observed by FAM-conjugated anti-digoxin. 4,6-Diamino-2-phenyl indole (DAPI) was used to stain the cell nuclei. Images were taken by an inverted fluorescence microscope (Mshot, MF52) at 488 nm.

### 2.6. Nuclear and Cytoplasmic RNA Fractionation Analysis

The RNA in nuclear and cytoplasmic of A549 and H1299 cells was isolated by Cytoplasmic & Nuclear RNA Purification Kit (Ambion, AM1921). And then, the expression levels of hsa_circ_0003176 were detected by RT-qPCR. GAPDH acted as the cytoplasm control, and U6 acted as the nuclear control.

### 2.7. Western Blot

The total protein was isolated by the RIPA lysis buffer (Solarbio) according to the instruction procedures. The protein was quantified by bicinchoninic acid (BCA) detection kit (Beyotime, China). And then, 40 *μ*g protein was loaded onto sodium dodecyl sulfate (SDS) gel, and then, the proteins were separated using the 10% SDS-PAGE. Subsequently, transfer protein bands onto the PVDF membranes (Millipore, USA). Then, the membrane was incubated with 5% skim milk at room temperature for 60 min. Then, the membranes were incubated by primary antibodies against anti-Rabbit RBM5 (Abcam, ab245646, 1 : 500) and anti-Rabbit GAPDH (Servicebio, GB11002, 1 : 1000) overnight at 4°C. After washing by PBS buffer for 3 times, the membranes were then incubated with the Goat anti-Rabbit IgG (Abcam, ab205718, 1 : 2000) at room temperature for 70 min. Finally, the protein bands were visualized by Gel imaging system (GE Healthcare) under ECL regent. The protein band was quantified using the Image J software (U. S. National Institutes of Health, Bethesda, Maryland, USA).

### 2.8. Cell Proliferation

Cell proliferation was detected by Cell Counting Kit-8 (CCK-8) Kit (C0042, Beyotime) according to the instruction manual. 2 × 10^3^ cells/well H1299 cells and A549 cells were plated in 96-well plates and subsequently grow in DMEM with 10% FBS for 24 h. 10 *μ*l Cell Count Kit-8 (CCK-8) solution was added to the 96-well plates after transfection, and cells underwent 24 h, 48 h, and 72 h incubation at 37°C in an incubator with 5% CO_2_. The absorbance (OD value) was detected by a spectrophotometer (Tecan, F50) at 450 nm.

### 2.9. Colony Formation Assay

Cell proliferation was also detected by the colony formation assay according to the instruction manual. Briefly, the treated NCSLC cells were seeded onto 6-well plates for 14 days, and the colonies were photographed and counted under the light microscope (Leica, Cat. DMI1).

### 2.10. Transwell Assay

Transwell chambers (0.8 *μ*m, Corning) containing the upper and lower chamber which separated by a polycarbonate membrane were seeded in the 24-well culture plate. 3 × 10^5^ cells/mL H1299 cells or A549 cells were prepared by serum-free medium. And then, the upper chamber containing 0.5% bovine serum albumin (BSA) was appended with 200 *μ*L/well. Subsequently, the lower chamber was appended with culture medium containing 10% FBS and cultured for 48 h. Subsequently, the cells in the lower chamber were fixed, dyed, and decolorized. Finally, the images were photographed under the light microscope (Leica, Cat. DMI1) and then counted by the Image J software (U. S. National Institutes of Health, Bethesda, Maryland, USA).

### 2.11. Wound-Healing Assay

The migration of the H1299 cells or A549 cells was detected by wound-healing assay. Briefly, the treated NSCLC cells at 80% confluence were scratched with a pipette tip and then incubated for 24 h. Images of cell migration were taken with a light microscope (Leica, Cat. DMI1) at 0 h and 24 h. Mobility/% = (0 h scratch width − 24 h scratch width)/0 h scratch width.

### 2.12. Luciferase Reporter Assay

The 3′UTR of RBM5 and hsa_circ_0003176 containing wild type and mutant sequences of binding sites was cloned into pmiGLO vector (Promega, USA) to generate pmiGLO-hsa_circ_0003176 vector and pmiGLO-RBM5 vector. And then, the pmiGLO-hsa_circ_0003176 vector or pmiGLO-RBM5 vector was cotransfected to NSCLC cells with miR-182-5p mimics or NC mimics, respectively. The luciferase was detected by Dual-Luciferase Assay System (Meilunbio, MA0518) at 48 h after the transfection following the instruction of manufacturer.

### 2.13. RNA Pull-Down Assay

The biotin-labeled probes for hsa_circ_0003176 were designed and synthesized by Sangon Biotech (Shanghai, China). The binding relationship between hsa_circ_0003176 and miR-182-5p was performed by the RNA pull-down assay. Briefly, the cells with were fixed, lysed, and centrifuged, and then, the supernatants were collected. Subsequently, the supernatants were incubated with the biotin-labeled probes for hsa_circ_0003176 overnight at room temperature. After that, the formaldehyde crosslinking was reversed by the lysis buffer and proteinase K. RNA was extracted by TRIzol reagent (Invitrogen, 15596-026). The supernatants were used as input, and the expression of hsa_circ_0003176 and miR-182-5p was detected by RT-qPCR.

### 2.14. In Vivo Experiments

Male BALB/c nude mice (SPF, 4 weeks old) were purchased at Zhuhai Baishitong Biotechnology Co., Ltd (License No.: SCXK (Guangdong) 2020-0051, Animal Certificate No. 44822700003250). After one week of adaptive feeding, subcutaneous xenograft experiments (*n* = 6 mice/group) and lung metastasis experiment (*n* = 5 mice/group) were carried out in nude mice. First, we constructed A549 cells with stable overexpressing hsa_circ_0003176. Briefly, the A549 cells were pretransfected with overexpressing hsa_circ_0003176 lentivirus, and then, the overexpressing hsa_circ_0003176 A549 cells was filtered with puromycin (MCE, USA) to get stable A549 cells. The A549 cells transfected with negative control lentivirus acted as negative control. The transfection efficiency was determined by RT-qPCR. For subcutaneous xenograft experiments, a total of 2 × 10^6^ A549 stable cells with or without overexpressing hsa_circ_0003176 were subcutaneously injected into the right armpit of male nude mice. The tumor volume and the weight of the nude mice were measured on days 3, 7, 14, 21, and 28 after subcutaneous xenograft, and tumor volume was calculated according to the formula volume = length × width2 × 0.5. At the end of feeding, mice were sacrificed with the tumor removed for weighing and histological analysis. For lung metastasis experiment, the nude mice were injected tail vein with a total of 2 × 10^6^ A549 stable cells with or without overexpressing hsa_circ_0003176. At the end of feeding (4 weeks), mice were sacrificed with the lung tissues removed for histological analysis. All the animal experiments were approved by the Ethics Committee of the University of Hong Kong-Shenzhen Hospital (approval number: HTSW201090924).

### 2.15. Histological Analysis

The collected tissues were routinely dehydrated, embedded, and cut into 3 *μ*m sections. For hematoxylin-eosin (H&E) staining, the sections were stained with hematoxylin and differentiation with hydrochloric acid alcohol. And then, ammonia water was used to immerse the sections for 10-30 s to return to the blue. And then, the sections were stained with eosin solution (Servicebio, G1004) and dehydrated with alcohol, xylene permeabilization and neutral gum sealing. After that, observation was performed under the light microscope (Leica, Cat. DMI1).

For Ki-67 immunohistochemistry, primary antibody anti-Ki67 (Proteintech, 21309-1-AP) was used to incubate the sections. After that, the sections were incubated with secondary antibody anti-rabbit horseradish peroxidase-conjugated antibody for 45 min. And then, the slides were stained using 3,3′diaminobenzidine (DAB) solution (Beyotime, P0202). Images were taken and analyzed with the light microscope (Leica, Cat. DMI1) and Image J software (U. S. National Institutes of Health, Bethesda, Maryland, USA), respectively. The expression level of Ki-67 was calculated as average optical density (AOD) = integrated option density (IOD)/area.

### 2.16. Statistical Analysis

Statistical analyses in the present study were detected using SPSS 22.0 software (IBM) and GraphPad Prism 8. Each study has at least three biological replicates. Data are shown as means ± standard deviation (SD). Pearson correlation coefficient analysis was used to evaluate correlations between genes. Differences between two groups and more than two groups were analyzed by Student's *t*-tests and one-way ANOVA assay. A value of *p* < 0.05 indicated a statistically significant difference.

## 3. Results

### 3.1. hsa_circ_0003176 Was Downregulated in NSCLC

**Figure 1:**
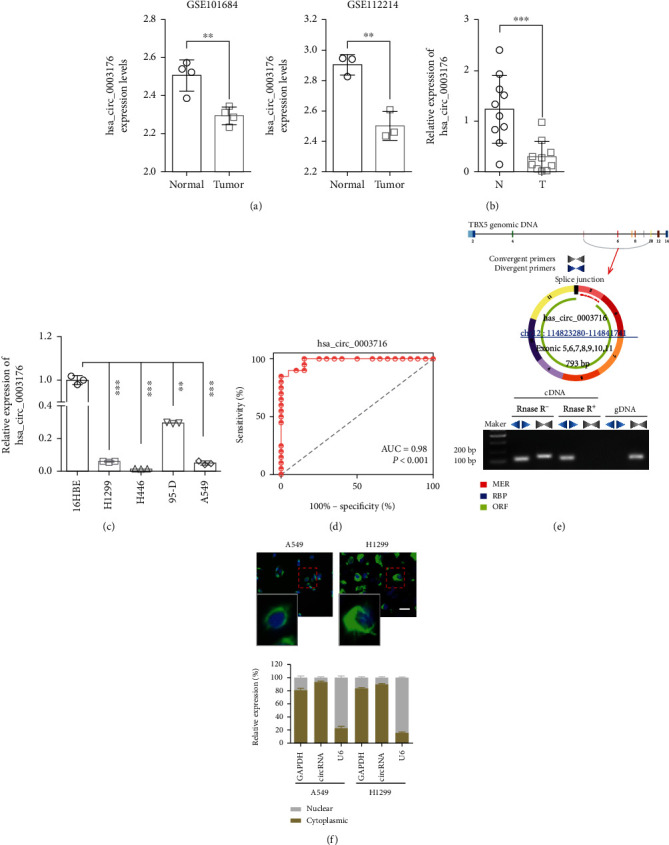
hsa_circ_0003176 was downregulated in both NSCLC tissues and cell lines. (a) The expression levels of hsa_circ_0003176 in NSCLC tissues and matched adjacent normal tissues based on GSE101684 datasets (https://www.ncbi.nlm.nih.gov/geo/query/acc.cgi?acc=GSE101684) and GSE112214 datasets (https://www.ncbi.nlm.nih.gov/geo/query/acc.cgi). The GSE101684 data sets contain four NSCLC lung samples and four matched adjacent normal samples (*N* = 4), whose sequencing platform is Arraystar Human CircRNA microarray V2. The GSE112214 data sets contain three NSCLC lung samples and three matched adjacent normal samples (*N* = 3), whose sequencing platform is Agilent-069978 Arraystar Human CircRNA microarray V1. (b) The expression of hsa_circ_0003176 was detected by RT-qPCR in 10 NSCLC tumor tissues and matched adjacent normal. (c) The expression of hsa_circ_0003176 was detected by RT-qPCR in lung cancer cell lines (including H1299, H446, 95-D, and A549) and normal control cell line (16HBE). *N* = 3. (d) ROC curve assay for hsa_circ_0003176 in 10 NSCLC. (e) Characteristics of hsa_circ_0003176 based on circBase and circRNADb databases and detected by RT-qPCR with divergent and convergent primers in cDNA and gDNA, respectively. (f) FISH and nuclear and cytoplasmic RNA fractionation assay were used to detect the localization of hsa_circ_0003176 in A549 and H1299 cells. U6 acts as nuclei control, GAPDH acts as cytoplasmic control, and cicrRNA indicated hsa_circ_0003176. Data was shown as mean ± SD. ^∗∗^*p* < 0.01 and ^∗∗∗^*p* < 0.001.

In order to investigate the role of hsa_circ_0003176 in NSCLC, we used GSE101684 containing four NSCLC lung samples and three matched adjacent normal samples (https://www.ncbi.nlm.nih.gov/geo/query/acc.cgi?acc=GSE101684) and GSE112214 datasets containing three NSCLC lung samples and three matched adjacent normal samples (https://www.ncbi.nlm.nih.gov/geo/query/acc.cgi) to analyze the hsa_circ_0003176 expression in NSCLC tissues and matched adjacent normal tissues. The result showed that hsa_circ_0003176 was significantly downregulated in NSCLC tissues compared with matched adjacent normal tissues (Figure 1(a)). In order to further confirm the expression of hsa_circ_0003176 in NSCLC, the expression of hsa_circ_0003176 was detected by RT-qPCR in NSCLC tissues, matched adjacent normal tissue, and cell lines. The results indicated that the expression of hsa_circ_0003176 was greatly downregulated in NSCLC lung tissues compared with matched adjacent normal tissues (Figure 1(b)). Meanwhile, we found that the expression of hsa_circ_0003176 was also significantly reduced in lung cancer cell lines (H1299, H446, 95-D, and A549) compared with 16HBE cells (Figure 1(c)). Further ROC curve indicated that the AUC reached at 98% with higher sensitivity and specificity (Figure 1(d)), suggesting that hsa_circ_0003176 has the certain diagnostic value for NSCLC. Furthermore, the data from circBase and circRNADb databases showed that hsa_circ_0003176 was transcribed from TBX5, which localized on chromosome 12 : 114823280-114841741 with 793 bp spliced mature circRNA length (Figure 1(e)). Furthermore, the target segment of hsa_circ_0003176 was amplified by divergent primers in cDNA regardless of RNase R treatment. On contrast, no target segment was observed amplified by convergent primers in cDNA when treatment with RNase R and amplified by divergent primers in gDNA (Figure 1(e)). In addition, FISH assay indicated that hsa_circ_0003176 was predominately localized to the cytoplasm of A549 and H1299 cells, and nuclear and cytoplasmic RNA fractionation analysis also confirmed the results (Figure 1(f)). These results suggested that hsa_circ_0003176 was downregulated in NSCLC and mainly located in the cytoplasm of NSCLC cells.

### 3.2. hsa_circ_0003176 Suppressed the Proliferation, Migration, and Invasion of NSCLC Cells

**Figure 2:**
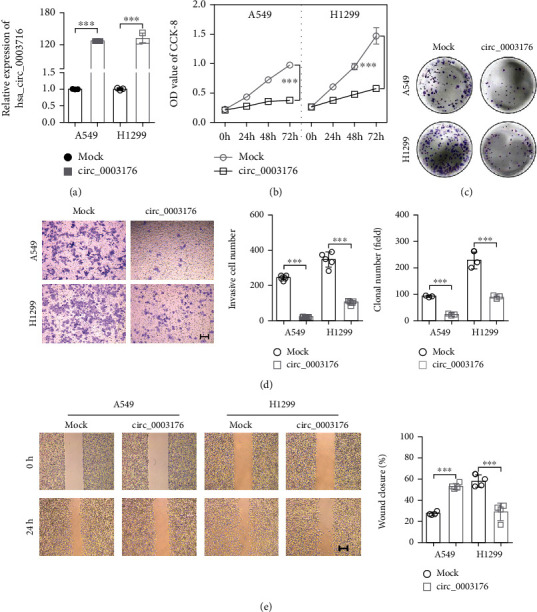
hsa_circ_0003176 suppressed the proliferation, migration, and invasion of NSCLC cells. (a) RT-qPCR was used to detect the expression of hsa_circ_0003176 in NSCLC cells (A549 and H1299) transfected with or without overexpressing hsa_circ_0003176 plasmid. (b) CCK-8 assay was used to detect the cell viability in NSCLC cells (A549 and H1299) transfected with or without overexpressing hsa_circ_0003176 plasmid. (c) Colony formation assay was used to detect the colony formation in NSCLC cells (A549 and H1299) transfected with or without overexpressing hsa_circ_0003176 plasmid. (d) Transwell assay was used to detect the cell invasion ability in NSCLC cells (A549 and H1299) transfected with or without overexpressing hsa_circ_0003176 plasmid. (e) The migration in NSCLC cells (A549 and H1299) transfected with or without overexpressing hsa_circ_0003176 plasmid was detected by wound-healing assay. Mock indicated negative control corresponding to hsa_circ_0003176 overexpression plasmid, and circ_0003176 indicated hsa_circ_0003176 overexpression plasmid. Data was shown as mean ± SD. *N* = 3 ~ 5. ^∗∗^*p* < 0.01 and ^∗∗∗^*p* < 0.001.

In order to explore the biological function of hsa_circ_0003176 on the progression of NSCLC, gain function of hsa_circ_0003176 was performed in A549 and H1299 cells by transfection with overexpressing hsa_circ_0003176 plasmid. RT-qPCR analysis showed that the expression of hsa_circ_0003176 was significantly upregulated in the hsa_circ_0003176 group compared with the mock group (Figure 2(a)). CCK-8 and colony formation assay indicated that the proliferation of both A549 and H1299 cells was inhibited when overexpression of hsa_circ_0003176 (Figures 2(b) and 2(c)). Transwell assay showed that the invasive cell number of both A549 and H1299 cells was notably reduced in the hsa_circ_0003176 group compared with that in the mock group (Figure 2(d)). Wound-healing assay showed that the wound closure of both A549 and H1299 cells was dramatically decreased in the overexpressing hsa_circ_0003176 group compared with that in the mock group (Figure 2(e)). These data suggested that hsa_circ_0003176 suppressed the proliferation, migration, and invasion of NSCLC cells.

### 3.3. hsa_circ_0003176 Inhibited NSCLC Growth and Metastasis In Vivo

**Figure 3:**
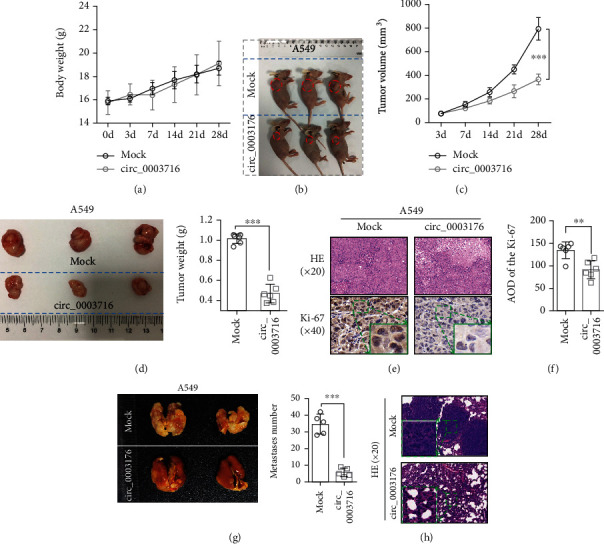
Overexpression of hsa_circ_0003176 inhibited NSCLC growth and metastasis in vivo. (a) Body weight was measured at 0, 3, 7, 14, 21, and 28 days after subcutaneous injection with overexpressing hsa_circ_0003176 stable A549 cells in nude mice. (b) The phenotypic characteristics of tumor volume in nude mice at 28 days after injection with overexpressing hsa_circ_0003176 stable A549 cells in subcutaneous of the right armpit. (c) The tumor volume was detected at 3, 7, 14, 21, and 28 days after subcutaneous injection with overexpressing hsa_circ_0003176 stable A549 cells in nude mice. (d) Tumor weight was detected at 28 days after subcutaneous injection with overexpressing hsa_circ_0003176 stable A549 cells in nude mice. (e) H&E staining and Ki-67 immunohistochemical staining were used to detect the structural changes and the expression of Ki-67 in tumor tissues at 28 days after subcutaneous injection with overexpressing hsa_circ_0003176 stable A549 cells in nude mice. (f) Statistical graph of Ki-67 expression levels in tumor tissues at 28 days after subcutaneous injection with overexpressing hsa_circ_0003176 stable A549 cells in nude mice. (g) The surface metastasis number in lung tissue at 28 days after tail vein injection with overexpressing hsa_circ_0003176 stable A549 cells in nude mice. (h) H&E staining was used to detect the number of lung metastasis nodules at 28 days after tail vein injection with overexpressing hsa_circ_0003176 stable A549 cells in nude mice. Data was shown as mean ± SD. *N* = 5 ~ 6, ^∗∗^*p* < 0.01, and ^∗∗∗^*p* < 0.001.

In order to further investigate the effect of hsa_circ_0003176 on NSCLC growth in vivo, a total of 2 × 10^6^ A549 stable cells with or without overexpressing hsa_circ_0003176 were subcutaneously injected into the right armpit of male nude mice. The result indicated that no significant difference was observed in body weight of nude mice between overexpressing hsa_circ_0003176 and mock groups during the experiment (Figure 3(a)). However, the tumor volume was significantly reduced in the overexpressing hsa_circ_0003176 group compared with that in the mock group (Figures 3(b) and 3(c)). And the tumor weight was also greatly reduced in the overexpressing hsa_circ_0003176 group compared with the mock group at the end of the experiment (Figure 3(d)). H&E staining showed that overexpressing hsa_circ_0003176 significantly reduced the inflammatory infiltration of tumor tissues compared with the mock group (Figure 3(e)). Ki-67 immunohistochemical staining assay showed that the Ki-67 expression was significantly downregulated by overexpressing hsa_circ_0003176 (Figures 3(e) and 3(f)), suggesting that overexpression of hsa_circ_0003176 inhibited NSCLC cell proliferation. In addition, to explore the effect of hsa_circ_0003176 on NSCLC metastasis in vivo, the nude mice were injected tail vein with a total of 2 × 10^6^ A549 stable cells with or without overexpressing hsa_circ_0003176. The result showed that the number of tumor metastasis on the surface of lung tissues in the overexpressing hsa_circ_0003176 group was significantly lower than that in the mock group (Figure 3(g)). H&E staining confirmed that the number of lung metastasis nodules was dramatically reduced in the overexpressing hsa_circ_0003176 group compared with the mock group (Figure 3(h)). These results indicated that overexpression of hsa_circ_0003176 inhibited NSCLC growth and metastasis.

### 3.4. hsa_circ_0003176 Suppressed the Proliferation, Migration, and Invasion of NSCLC Cells via Sponging miR-182-5p

**Figure 4:**
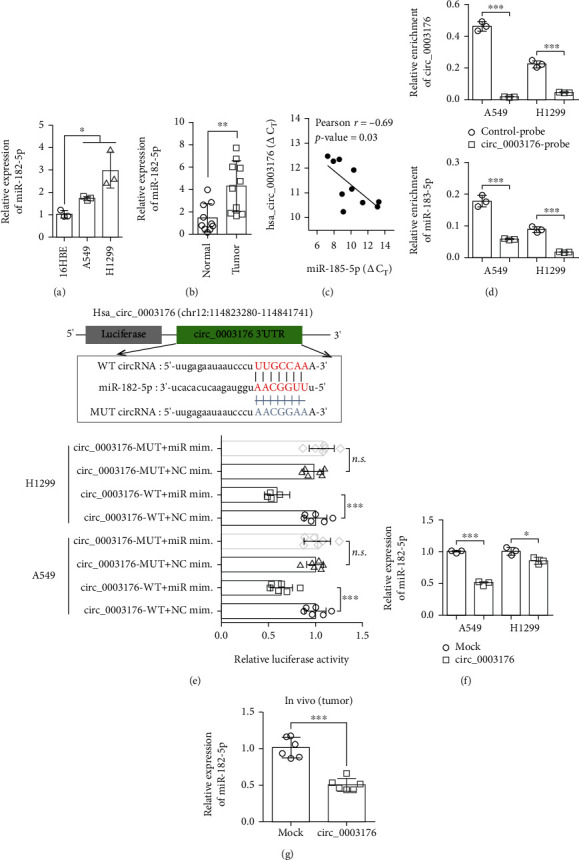
Hsa_circ_0003176 suppressed miR-185-5p expression by sponging miR-182-5p. (a) The expression of miR-182-5p in NSCLC cell lines (H1299 and A549) and normal control cell line (16HBE) was detected by RT-qPCR. *N* = 3. (b) The expression of miR-182-5p was detected by RT-qPCR in 10 NSCLC tissues and matched adjacent normal tissues. (c) Pearson correlation coefficient was used to analyze the correlation between the RBM5 expression and miR-182-5p expression in 10 NSCLC tissues. (d) RNA pull-down assay was used to detect the binding relationship between miR-182-5p and hsa_circ_0003176. *N* = 3. (e) The dual-luciferase reporter assay was used to determine the direct interaction between miR-182-5p and hsa_circ_0003176. NC mim. indicated negative control mimics, and miR mim. indicated miR-182-5p mimics. *N* = 6. (f) RT-qPCR was used to evaluate the expression of miR-182-5p in NSCLC cells (H1299 and A549) transfected with or without hsa_circ_0003176 overexpression plasmid. *N* = 3. (g) RT-qPCR was used to evaluate the effect of hsa_circ_0003176 on miR-182-5p expression in vivo. *N* = 6. Data was shown as mean ± SD. ^∗^*p* < 0.5, ^∗∗^*p* < 0.01, and ^∗∗∗^*p* < 0.001.

Several studies have proved that circRNAs affected miRNA activity by acting as miRNA “sponge” [15, 16]. To explore regulatory functions of hsa_circ_0003176 at posttranscriptional level, we used CircInteractome to predict the potential binding miRNA of hsa_circ_0003176. We found that miR-182-5p showed the highest binding score (Table S1). Previous studies have indicated that miR-182-5p is an oncogenic gene in non-small-cell lung cancer [17, 18]. RT-qPCR results also indicated that the expression of miR-182-5p was significantly upregulated in both NSCLC cell lines and tumor tissues compared with their corresponding controls (Figures 4(a) and 4(b)). Pearson correlation coefficient analysis showed that the expression of hsa_circ_0003176 and miR-182-5p was negatively correlated in 10 NSCLC tissues (Figure 4(c)). RNA pull-down assay showed that a specific enrichment of hsa_circ_0003176 and miR-182-5p was detected by qRT-PCR in the hsa_circ_0003176 probe group compared with control probe in A549 cells and H1299 cells (Figure 4(d)), suggesting the interaction between hsa_circ_0003176 and miR-182-5p in NSCLC cells. Moreover, dual-luciferase reporter assays were used to verify direct interaction between hsa_circ_0003176 and miR-182-5p. Cotransfection cells with hsa_circ_0003176-WT vector and miR-182-5p mimics led to significantly reduced relative luciferase activity, while co-transfection with hsa_circ_0003176-MUT vector and miR-182-5p mimics induced nonsignificant differences (Figure 4(e)), suggesting that hsa_circ_0003176 interacts directly with miR-182-5p in NSCLC. In addition, the expression of miR-182-5p affected by hsa_circ_0003176 both in vitro and in vivo was detected. The results indicated that the expression of miR-182-5p was dramatically decreased in A549 and H1299 cells (Figure 4(f)) and in subcutaneous xenograft (Figure 4(g)) with hsa_circ_0003176 overexpression, indicating that hsa_circ_0003176 could negatively regulate miR-182-5p expression. Taken together, the above results showed that hsa_circ_0003176 interacted directly with miR-182-5p to inhibit its expression.

**Figure 5:**
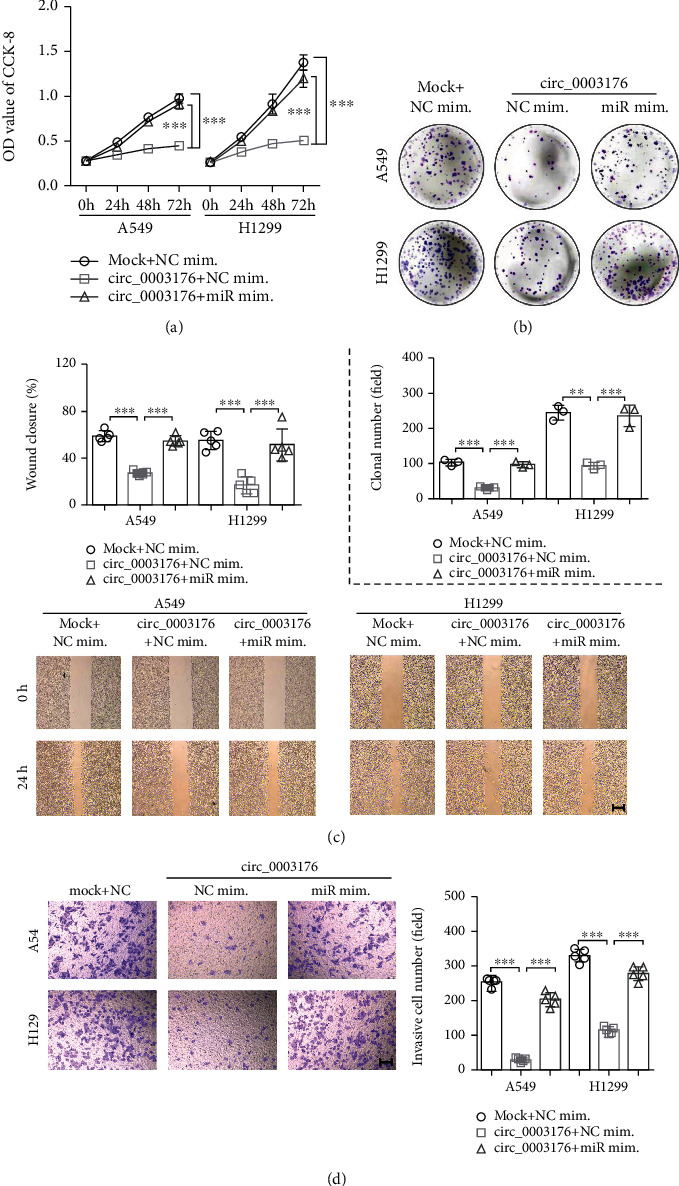
hsa_circ_0003176 suppressed the proliferation, migration, and invasion of NSCLC cells by downregulating miR-182-5p expression. (a) The viability of NSCLC cell lines (A549 and H1299) transfected with hsa_circ_0003176 overexpression plasmid alone or cotransfected with miR-182-5p mimics was detected using CCK-8 assay. (b) The colony formation ability of NSCLC cell lines (A549 and H1299) transfected with hsa_circ_0003176 overexpression plasmid alone or cotransfected with miR-182-5p mimics was detected using colony formation assay. (c) The migration ability of NSCLC cell lines (A549 and H1299) transfected with hsa_circ_0003176 overexpression plasmid alone or cotransfected with miR-182-5p mimics was detected using wound-healing assay. (d) The invasion ability of NSCLC cell lines (A549 and H1299) transfected with hsa_circ_0003176 overexpression plasmid alone or cotransfected with miR-182-5p mimics was detected using transwell invasion assay. Data was shown as mean ± SD. *N* = 3 ~ 5, ^∗∗^*p* < 0.01, and ^∗∗∗^*p* < 0.001.

To explore further whether hsa_circ_0003176 suppressed the proliferation, migration, and invasion of NSCLC cells via regulating miR-182-5p, the NSCLC cell lines (A549 and H1299) were transfected with hsa_circ_0003176 overexpression plasmid alone or cotransfected with miR-182-5p mimics. CCK-8 and colony formation assay indicated that overexpressing miR-182-5p could reduce the proliferation inhibition to both A549 and H1299 cells caused by overexpression of hsa_circ_0003176 (Figures 5(a) and 5(b)). Wound-healing assay showed that the migration inhibited by overexpressing hsa_circ_0003176 in both A549 and H1299 cells was also alleviated when treatment with miR-182-5p mimics (Figure 5(c)). Transwell assay showed that the reduced invasive cell number of both A549 and H1299 cells in the hsa_circ_0003176 group was partially increased when transfection with miR-182-5p mimics (Figure 5(d)). These data suggested that hsa_circ_0003176 suppressed the proliferation, migration, and invasion of NSCLC cells by downregulating miR-182-5p expression.

### 3.5. hsa_circ_000 3176 Enhanced RBM5 Expression via Regulating miR-182-5p

**Figure 6:**
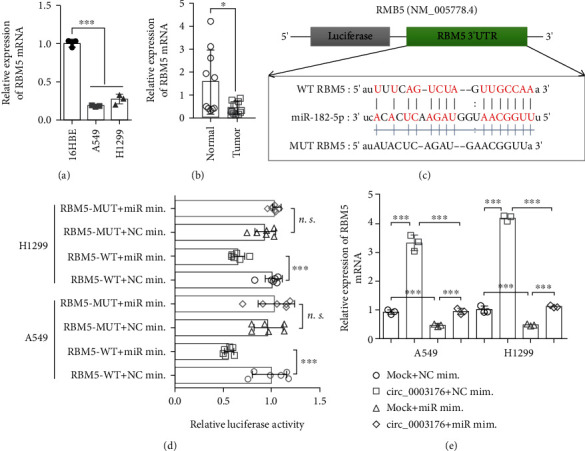
hsa_circ_000 3176 regulates RBM5 expression by regulating miR-182-5p. (a) The mRNA expression of RBM5 in NSCLC cell lines (H1299 and A549) and normal control cell line (16HBE) was detected by RT-qPCR. *N* = 3. (b) The mRNA expression of RBM5 was detected by RT-qPCR in 10 NSCLC tissues and matched adjacent normal tissues. (c) Putative binding sites of miR-182-5p with respect to RBM5 were predicated. (d) The luciferase activity of RBM5 in the NSCLC cells after cotransfection with miR-182-5p mimics or NC mimics and RBM5-WT or RBM5-MUT. (e) RT-qPCR was used to detect the expression of RBM5 in NSCLC cell lines (A549 and H1299) transfected with hsa_circ_0003176 overexpression plasmid alone or cotransfected with miR-182-5p mimics. Data was shown as mean ± SD. *N* = 3 ~ 6, ^∗^*p* < 0.5, and ^∗∗∗^*p* < 0.001.

circRNAs can regulate the expression of the target genes of miRNAs by acting as a ceRNA to sponge microRNA [19]. We predicted 132 potential common target genes of miR-182-5p by RITA, miRmap, miRanda, and TargetScan databases, including RBM5 (Figure S1a). RBM5 (RNA-binding protein 5) has been confirmed as a tumor suppressor in different cancers, including lung cancer [20]. Therefore, RBM5 was identified as potential targets of miR-182-5p for further study. We further verified the expression level of RBM5 in NSCLC and its relationship with miR-182-5p based on bioinformatics and RT-qPCR. The data from GEPIA database indicated the expression of RBM5 was downregulated in LUAD tumor tissues compared with control tissues (Figure S1b). And highly expression of RBM5 showed better overall survival (Figure S1c). Furthermore, the expression of RBM5 was significantly decreased in both NSCLC cell lines and tumor tissues compared with control (Figures 6(a) and 6(b)). The above results suggested that the RBM5 was significantly downregulated in NSCLC and might be a target gene of miR-182-5p.

To investigate the directly interaction between miR-182-5p and RBM5, dual-luciferase reporter gene detection was performed with wild-type and mutant RBM5–3′ untranslated region (UTR) vectors incorporating miR-182-5p binding sites (RBM5-wild-type vector and RBM5-mutant-type vector) (Figure 6(c)). The result indicated that the relative luciferase activity was dramatically decreased when cotransfection with miR-182-5p mimics and RBM5-WT vector while was not changed when cotransfection with miR-182-5p mimics and RBM5-MUT vector (Figure 6(d)), clearly indicating miR-182-5p interacts directly with RBM5 in NSCLC. In addition, we found that miR-182-5p overexpression significantly decreased the RBM5 expression in A549 cells and H1299 cells (Figure 5(e) and Figure S2A), whereas silencing miR-185-5p dramatically increased RBM5 expression (Figure S2B). Meanwhile, we found that hsa_circ_0003176 overexpression markedly upregulated the RBM5 expression, while the phenomena could be partially reversed by overexpression miR-182-5p (Figure 5(e) and Figure S2a). Taken together, these results indicated that hsa_circ_0003176 enhanced RBM5 expression via inhibiting miR-182-5p expression in NSCLC cells.

### 3.6. hsa_circ_0003176 Suppressed the Proliferation, Migration, and Invasion of NSCLC Cells by Upregulating RBM5 Expression

**Figure 7:**
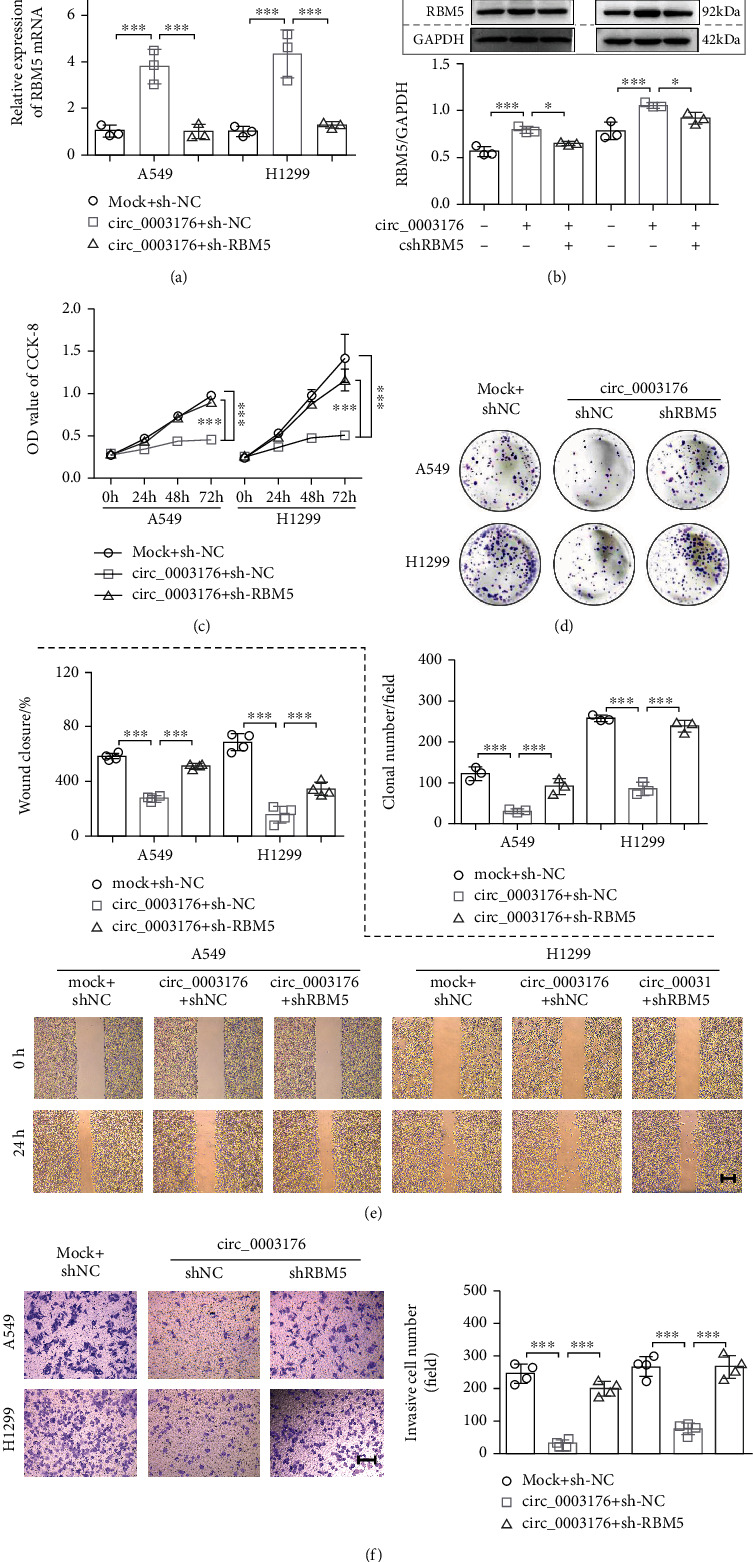
hsa_circ_0003176 suppressed the proliferation, migration, and invasion of NSCLC cells by upregulating RBM5 expression. (a) The mRNA expression of RBM5 in NSCLC cell lines (A549 and H1299) transfected with hsa_circ_0003176 overexpression plasmid alone or cotransfected with shRBM5 plasmid was detected using RT-qPCR. (b) The protein expression of RBM5 NSCLC cell lines (A549 and H1299) transfected with hsa_circ_0003176 overexpression plasmid alone or cotransfected with shRBM5 plasmid was detected using western blotting. (c) The viability of NSCLC cell lines (A549 and H1299) transfected with hsa_circ_0003176 overexpression plasmid alone or cotransfected with shRBM5 plasmid was detected using CCK-8 assay. (d) The colony formation ability of NSCLC cell lines (A549 and H1299) transfected with hsa_circ_0003176 overexpression plasmid alone or cotransfected with shRBM5 plasmid was detected using colony formation assay. (e) The migration ability of NSCLC cell lines (A549 and H1299) transfected with hsa_circ_0003176 overexpression plasmid alone or cotransfected with shRBM5 plasmid was detected using wound-healing assay. (f) The invasion ability of NSCLC cell lines (A549 and H1299) transfected with hsa_circ_0003176 overexpression plasmid alone or cotransfected with shRBM5 plasmid was detected using transwell invasion assay. Mock indicated negative control corresponds to hsa_circ_0003176 overexpression plasmid, and shNC indicated negative control corresponds to shRBM5 plasmid. Data was shown as mean ± SD. *N* = 3 ~ 5, ^∗∗^*p* < 0.01, and ^∗∗∗^*p* < 0.001.

In order to know whether hsa_circ_0003176 participated in the regulation of NSCLC cell proliferation, migration, and invasion by regulating RBM5 expression, the designed RBM5 shRNA (shRNA#1, shRNA#2, and shRNA#3) was used to silence the expression of RBM5 in both A549 and H1299 cells. The results indicated that all of the shRNAs for RBM5 significantly downregulated the expression of RBM5, and both shRNA#1 and shRNA#2 showed good interference effect (Figure S3). Thus, one of them (shRNA#1) was selected for further study. Subsequently, the NSCLC cell lines (A549 and H1299) were transfected with hsa_circ_0003176 overexpression plasmid alone or cotransfected with shRBM5 plasmid, and RT-qPCR and western blot assay showed that transfection of sh-RBM5 plasmid can reduce the mRNA and protein expression of RBM5 in A549 and H1299 cells overexpressing hsa_circ_0003176 (Figures 7(a) and 7(b)). CCK-8 and colony formation assay indicated that silencing RBM5 could reduce the proliferation inhibition to both A549 and H1299 cells caused by overexpression of hsa_circ_0003176 (Figures 7(c) and 7(d)). Wound healing and transwell invasion assay showed that the migration ability and invasion ability inhibited by overexpressing hsa_circ_0003176 in both A549 and H1299 cells could be partially alleviated by silencing RBM5 (Figures 7(e) and 7(f)). Taken together, these results indicated that hsa_circ_0003176 suppressed the proliferation, migration, and invasion of NSCLC cells by upregulating RBM5 expression.

## 4. Discussion

Lung cancer is one of the common malignant tumors, among which NSCLC accounts for about 80% of the incidence of lung cancer [21]. Most of the patients are found in the advanced stage, and the prognosis is very poor due to the difficulty of the screening and diagnosis [22]. At present, the main treatments are surgery, radiotherapy, chemotherapy, and targeted therapy, but the overall cure rate is low, and there are many adverse reactions and defects that seriously affect the quality of life [23]. Numerous studies have shown that circRNAs play important roles in the NSCLC [24]. However, the biological functions and regulatory mechanisms of most circRNAs in NSCLC remain still unknown. Therefore, exploring the roles and mechanisms of circRNAs in NSCLC is helpful for early diagnosis, targeted therapy, and improvement of prognosis of NSCLC. In the present study, we found that hsa_circ_0003176 suppressed the NSCLC progression via regulating miR-182-5p/RBM5 axis, and these findings indicated that hsa_circ_0003176 might be a novel molecular target for NSCLC treatment.

Circular RNAs (circRNAs) are a class of endogenously conserved noncoding RNAs that have been shown to be involved in transcriptional and posttranscriptional gene regulation, thereby regulating the occurrence and development of various diseases, including NSCLC [8, 25, 26]. For example, Zhang et al. demonstrated that the highly expression of circRNA_0001946 was positively correlated with lower TNM stage, less lymph node metastasis [27]. Yang et al. indicated that circRNA_001846 showed 78.2% sensitivity and 81.1% specificity values with an AUC value of 0.872, suggesting that circRNA_001846 might be a potential diagnostic biomarker for NSCLC [28]. Ishola et al. demonstrated that overexpression of circRNA C190 promoted NSCLC by modulating the EGFR/ERK pathway [29]. In the present study, we identified hsa_circ_0003176 showed typical characteristics of circRNAs, which was downregulated in both NSCLC tissues and cell lines. Functionally, we demonstrated that overexpression of hsa_circ_0003176 suppressed the proliferation, migration, and invasion of NSCLC cells in vitro and inhibited NSCLC tumor growth and metastasis in vivo. These results suggested that hsa_circ_0003176 acted as a tumor suppressor gene in NSCLC progression.

It has been reported in the literature that circRNAs can act as miRNA sponges to regulate the expression levels of miRNAs and downstream target genes [30, 31]. In NSCLC, several studies indicated that circRNA could act as sponge of miRNA that regulated the downstream gene expression. For instance, circ-MYBL2 inhibited cell proliferation and apoptosis by sponging oncogenic miR-28 in NSCLC [32]. hsa_circ_0005909 was proved to promote NSCLC growth, metastasis, and drug resistance by regulating SOX4 expression via sponging miR-338-3p [33]. In the present study, we proved that hsa_circ_0003176 regulated RBM5 expression via sponging miR-182-5p. Zhang et al. showed that miR-182-5p was significantly increased in NSCLC tumor tissues and was correlated with tumor size and cell proliferation, suggesting that miR-182-5p played an oncogenic role in NSCLC [34]. Yang et al. found that miR-182-5p was upregulated in the NSCLC tissues and promoted the NSCLC metastasis and epithelial-mesenchymal transition (EMT) by regulating EPAS1 [18]. In consistent with these results, we demonstrated that hsa_circ_0003176 suppressed the proliferation, migration, and invasion of NSCLC cells by downregulating miR-182-5p expression.

In addition, it has been demonstrated that RBM5 acts as tumor suppressor in different cancers. Compared with normal tissues, RBM5 was significantly lower in medulloblastoma and RBM5 overexpression repressed cell proliferation and migration in vitro [35]. Zilun et al. demonstrated that long noncoding RNA LINP1 (LncRNA LINP1) inhibited apoptosis and promoted proliferation by repressing RBM5 in gastric cancer cells, suggesting that RBM5 plays a tumor suppressor role in gastric cancer [36]. Jamsai et al. and Sutherland indicated that act as a tumor suppressor in the lung by decreasing adenocarcinoma nodule numbers and tumor size [20, 37]. Su et al. showed that RBM5 overexpression improves the antitumor effect by inducing autophagy in human lung adenocarcinoma cells [38]. In consistent with these findings, we demonstrated that hsa_circ_0003176 suppressed the proliferation, migration, and invasion of NSCLC cells by upregulating RBM5 expression. Taken together, we demonstrated that hsa_circ_0003176 suppresses the progression of NSCLC via regulating miR-182-5p/RBM5 axis.

In this study, our results demonstrated that hsa_circ_0003176 inhibited the growth and metastasis of NSCLC in vitro and in vivo and elucidated that hsa_circ_0003176 regulated the malignant biological functions of NSCLC cells by targeting the expression of RBM5 through sponge adsorption of miR-182-5p in vitro, but hsa_circ_0003176 inhibiting NSCLC progression by regulating miR-182-5p/RBM5 axis has not been verified in animals. In the next step, we will further validate this result in animals, expand clinical samples to verify the clinical significance of hsa_circ_0003176/miR-182-5p/RBM5 signaling axis, and explore the underlying molecular mechanism of hsa_circ_0003176 dysfunction in NSCLC.

## 5. Conclusion

Our results indicated that hsa_circ_0003176 suppressed the progression of NSCLC via regulating RBM5 expression by sponging miR-182-5p. These findings provided potential therapeutic targets to develop new strategy for NSCLC treatment.

## Data Availability

The circRNA expression profile was investigated in GSE101684 (https://www.ncbi.nlm.nih.gov/geo/query/acc.cgi?acc=GSE101684) and GSE112214 (https://www.ncbi.nlm.nih.gov/geo/query/acc.cgi?acc=GSE112214) data sets from GEO database.

## Supplementary Materials

Supplementary Materials Supplementary 1. Figures S1: RBM5 might be a target of miR-182-5p. (a) Venn diagram showed that the potential binding target genes of miR-182-5p were predicted by RITA, miRmap, miRanda, and TargetScan databases. (b) Expression of RBM5 in LUAD tumor tissues from the GEPIA database with 483 tumor samples and 347 normal tissue samples. Red squares represent lung cancer tissue, and gray squares represent normal lung tissue. (c) Overall survival of RBM5 in NSCLS patients. Data was shown as mean ± SD. ^∗^*p* < 0.05. Supplementary 2. Figures S2: (a) RT-qPCR was used to detect the expression of miR-182-5p in NSCLC cell lines (A549 and H1299) transfected with hsa_circ_0003176 overexpression plasmid alone or cotransfected with miR-182-5p mimics. (b) RT-qPCR was used to detect the expression of miR-182-5p and RBM5 in NSCLC cells transfected with miR-185-5p inhibitor or NC inhibitor. Data was shown as mean ± SD. *N* = 3, ^∗∗^*p* < 0.01, and ^∗∗∗^*p* < 0.001. Supplementary 3. Figures S3: RT-qPCR was used to detect expression of RBM5 in both A549 and H1299 cells by transfecting the shRNA of RBM5 (shRNA#1-3). Data was shown as mean ± SD. *N* = 3, ^∗^*p* < 0.5, ^∗∗^*p* < 0.01, and ^∗∗∗^*p* < 0.001. Supplementary 4. Table S1: the potential binding miRNA of hsa_circ_0003176 was predicted by the online website CircInteractome.
