# Supplementary material for: hsa_circ_0003176 Suppresses the Progression of Non-Small-Cell Lung Cancer via Regulating miR-182-5p/RBM5 Axis
Source: Dis Markers. 2022 Sep 23;2022:8402116. doi: 10.1155/2022/8402116 (PMC9525747; doi:10.1155/2022/8402116)
Supplement: Supplementary Materials — Supplementary 1. Figures S1: RBM5 might be a target of miR-182-5p. (a) Venn diagram showed that the potential binding target genes of miR-182-5p were predicted by RITA, miRmap, miRanda, and TargetScan databases. (b) Expression of RBM5 in LUAD tumor tissues from the GEPIA database with 483 tumor samples and 347 normal tissue samples. Red squares represent lung cancer tissue, and gray squares represent normal lung tissue. (c) Overall survival of RBM5 in NSCLS patients. Data was shown as mean ± SD. ∗p < 0.05. Supplementary 2. Figures S2: (a) RT-qPCR was used to detect the expression of miR-182-5p in NSCLC cell lines (A549 and H1299) transfected with hsa_circ_0003176 overexpression plasmid alone or cotransfected with miR-182-5p mimics. (b) RT-qPCR was used to detect the expression of miR-182-5p and RBM5 in NSCLC cells transfected with miR-185-5p inhibitor or NC inhibitor. Data was shown as mean ± SD. N = 3, ∗∗p < 0.01, and ∗∗∗p < 0.001. Supplementary 3. Figures S3: RT-qPCR was used to detect expression of RBM5 in both A549 and H1299 cells by transfecting the shRNA of RBM5 (shRNA#1-3). Data was shown as mean ± SD. N = 3, ∗p < 0.5, ∗∗p < 0.01, and ∗∗∗p < 0.001. Supplementary 4. Table S1: the potential binding miRNA of hsa_circ_0003176 was predicted by the online website CircInteractome. [file 8402116.f1.docx]

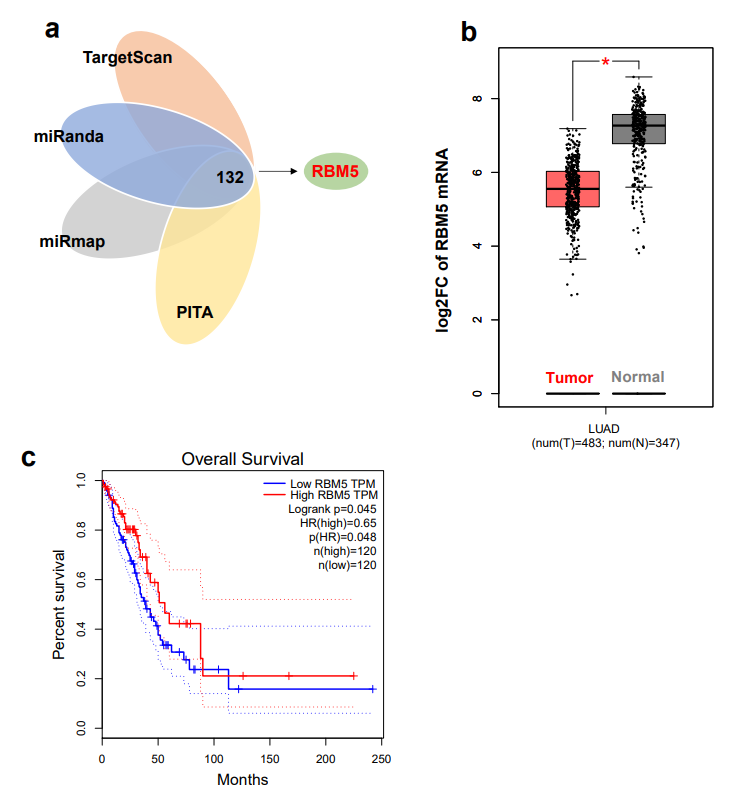


*Supplementary1*. Figures S1: RBM5 might be a target of miR-182-5p. (a) Venn diagram showed that the potential binding target genes of miR-182-5p were predicted by RITA, miRmap, miRanda and TargetScan databases. (b) Expression of RBM5 in LUAD tumor tissues from the GEPIA database with 483 tumor samples and 347 normal tissue samples. Red squares represents lung cancer tissue, and gray squares represents normal lung tissue. (c) Overall survival of RBM5 in NSCLS patients. Data was shown as mean±SD. *p<0.05.


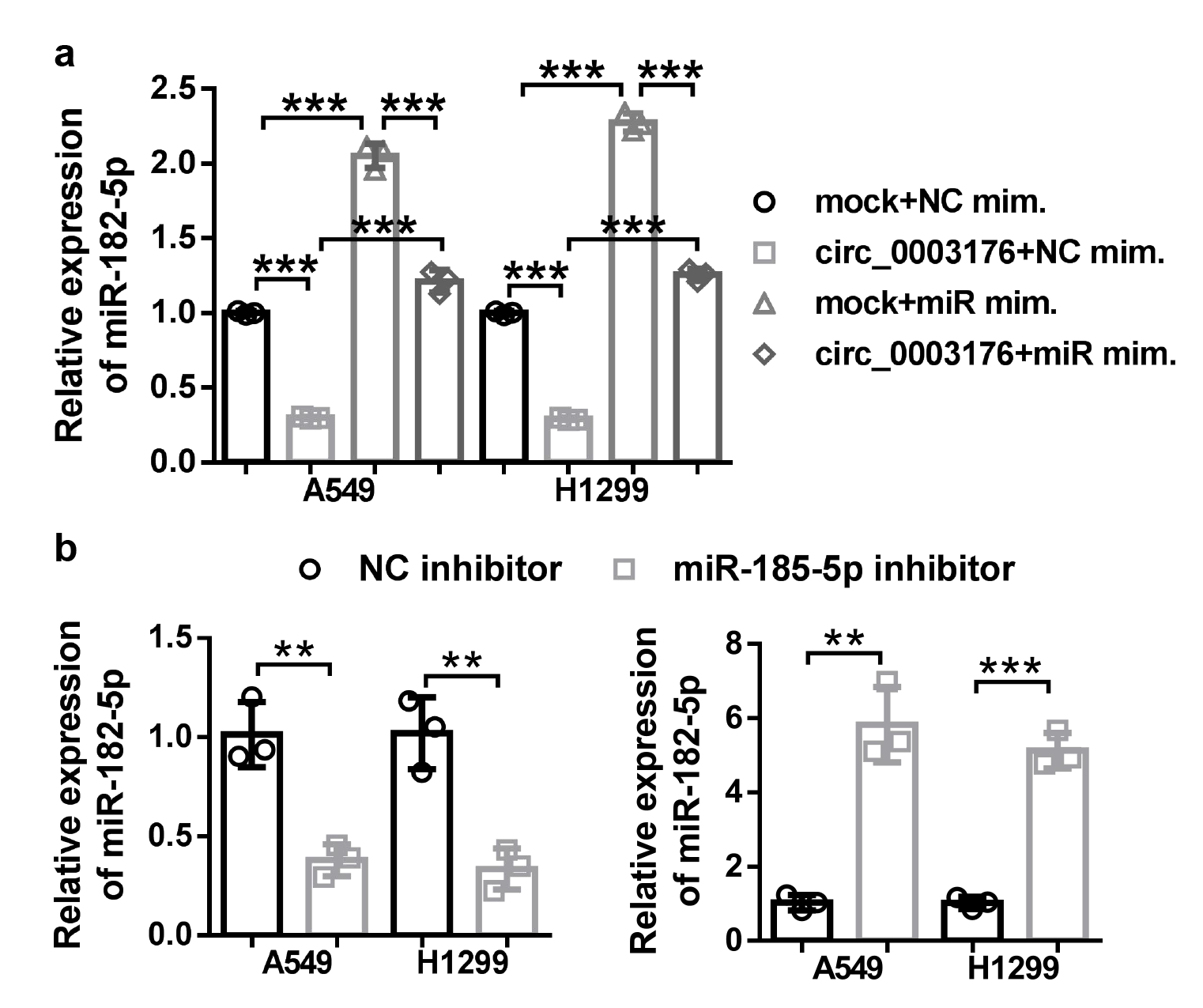


*Supplementary2*. Figures S2: a. RT-qPCR was used to detect the expression of miR-182-5p in NSCLC cell lines (A549 and H1299) transfected with hsa_circ_0003176 overexpression plasmid alone or co-transfected with miR-182-5p mimics. b. RT-qPCR was used to detect the expression of miR-182-5p and RBM5 in NSCLC cells transfected with miR-185-5p inhibitor or NC inhibitor. Data was shown as mean±SD. N = 3, **p<0.01, ***p<0.001.


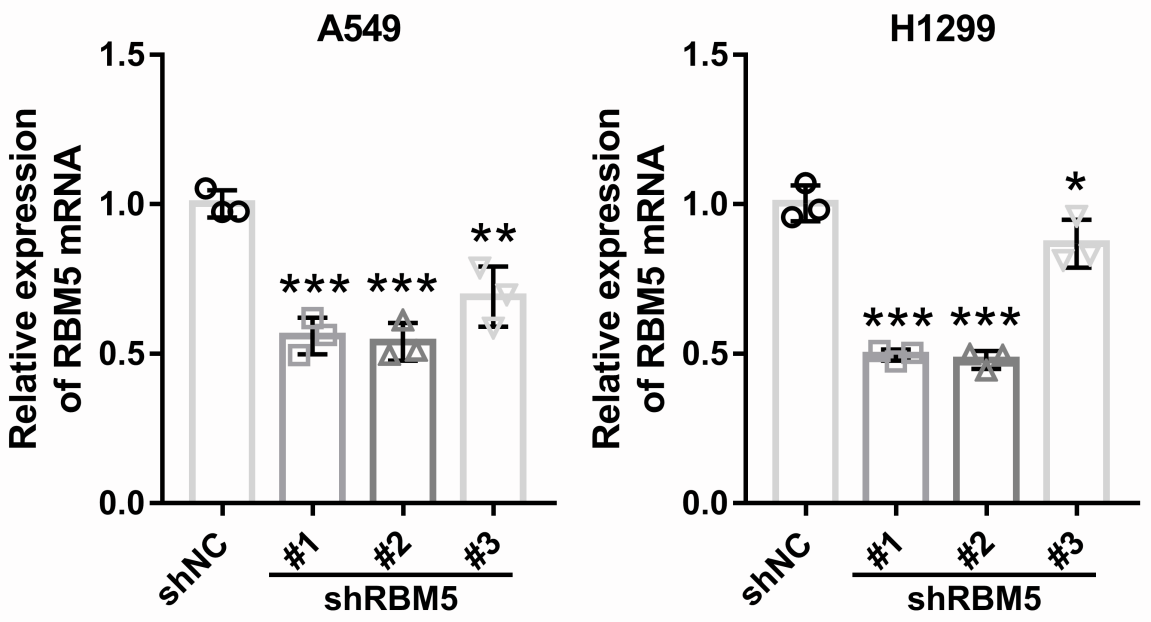


*Supplementary3*. Figures S3: RT-qPCR was used to detect expression of RBM5 in both A549 and H1299 cells by transfected by the shRNA of RBM5 (shRNA#1-3). Data was shown as mean±SD. N = 3, *p<0.5, **p<0.01, ***p<0.001.

*Supplementary4.* Table S1 The potential binding miRNAs of hsa_circ_0003176 was predicted by the online website CircInteractome.

| **Name** | **Score** | **Score percentile** |
| --- | --- | --- |
| hsa-miR-182-5p | -0.298 | 98 |
| hsa-miR-1253 | -0.221 | 98 |
| hsa-miR-1299 | -0.16 | 92 |
| hsa-miR-224 | -0.119 | 91 |
| hsa-miR-490-5p | -0.135 | 90 |
| hsa-miR-581 | -0.129 | 89 |
| hsa-miR-885-5p | -0.098 | 89 |
| hsa-miR-558 | -0.177 | 88 |
| hsa-miR-874 | -0.164 | 87 |
| hsa-miR-876-3p | -0.148 | 87 |
| hsa-miR-526b | -0.103 | 87 |
| hsa-miR-146b-3p | -0.159 | 85 |
| hsa-miR-873 | -0.09 | 83 |
| hsa-miR-769-3p | -0.129 | 81 |
| hsa-miR-582-3p | -0.109 | 81 |
| hsa-miR-450b-3p | -0.126 | 80 |
| hsa-miR-1234 | -0.142 | 78 |
| hsa-miR-933 | -0.227 | 77 |
| hsa-miR-370 | -0.12 | 77 |
| hsa-miR-637 | -0.131 | 76 |
| hsa-miR-494 | 0.047 | 72 |
| hsa-miR-296-5p | -0.193 | 71 |
| hsa-miR-146b-3p | -0.096 | 71 |
| hsa-miR-516b | -0.07 | 70 |
